# The Biosocial Microbiome: Gender Identity, Geography, and Mucosal Microbial Phenotypes

**DOI:** 10.21203/rs.3.rs-8368158/v1

**Published:** 2026-01-13

**Authors:** Vanessa Van Doren, S. Smith, Cassie Grimsley-Ackerley, Jadah Keith, Robert Arthur, Henry Claussen, Phillip Murray, Vin Tangpricha, Yijuan Hu, Chang Su, Mengyu He, Colleen Kelley

**Affiliations:** The Hope Clinic of the Emory Vaccine Center; The Hope Clinic of the Emory Vaccine Center; The Hope Clinic of the Emory Vaccine Center

## Abstract

**Background:**

Transgender women (TGW) experience unique hormonal contexts and high HIV incidence, yet the mucosal microbiome among TGW remains understudied. Sex hormones and geography may shape microbial composition, but the relative contributions of gender identity, feminizing hormone therapy (FHT), and location to mucosal microbial phenotypes among key populations such as TGW are unknown.

**Methods:**

We conducted a multi-site study of cisgender men who have sex with men (MSM) and TGW using FHT, both without HIV, in Atlanta, Georgia, USA (n = 58; 25 TGW, 33 MSM) and Bangkok, Thailand (n = 147; 97 TGW, 50 MSM), using cross-sectional sampling (n = 205). We also conducted longitudinal sampling in TGW (n = 21) pre/post FHT initiation. Rectal mucosal swabs were collected from all participants with optional neovaginal sampling in TGW. Microbiota composition was analyzed using 16S rRNA sequencing, and associations with gender identity, geography, and serum estradiol and testosterone concentrations were assessed using linear decomposition modeling (LDM)(1) and BOUTH analysis(2).

**Results:**

Rectal microbiota differed significantly by both gender identity and geography via LDM and BOUTH analyses. TGW exhibited enrichment of estrogen-metabolizing taxa across sites, while MSM showed *Prevotellaceae* enrichment in Atlanta but not Bangkok. Alpha diversity varied by location but not gender identity. Neovaginal microbiota differed markedly from rectal composition, showing enrichment of skin- and gut-associated taxa (e.g., *Prevotella, Peptostreptococcus, Porphyromonas*) and anaerobic taxa associated with HIV seroconversion. Longitudinal analysis revealed no significant rectal microbiota shifts after short-term FHT initiation, possibly reflecting subtherapeutic hormone exposure.

**Conclusions:**

These findings underscore the need to consider gender identity as a complex biosocial phenotype in HIV prevention and highlight the potential role of mucosal microbiota in shaping HIV vulnerability in TGW.

## BACKGROUND

The intestinal microbiota is a critical determinant of mucosal immune function, epithelial barrier integrity, and metabolic signaling(3–5). Rectal mucosal communities, in particular, influence susceptibility to enteric and sexually transmitted infections, including HIV, by modulating local inflammation and availability of viral target cells(6). Among cisgender men who have sex with men (MSM), receptive anal intercourse (RAI) is associated with distinct rectal microbiota features, often characterized by *Prevotella* enrichment and reduced microbial diversity, that have been linked to mucosal immune activation and HIV acquisition(7, 8). However, little is known about the rectal microbiota in transgender women, a population with disproportionately high HIV incidence and frequent use of feminizing hormone therapy that could influence microbiota composition (FHT).

Sex hormones are emerging as key regulators of gut microbial ecology, with growing evidence from experimental and human studies that both androgens and estrogens shape microbial community composition through multiple pathways, including modulation of bile acid metabolism, epithelial barrier function, and mucosal immune tone(9–12). The gut microbiota can also metabolize and recycle both androgens and estrogens through microbial β-glucuronidase activity and enterohepatic recirculation(13, 14). These bidirectional interactions have been implicated in cardiometabolic, autoimmune, and oncologic outcomes that differ by sex, yet their relevance to mucosal sites implicated in HIV transmission or among people who use gender affirming hormone therapy remains poorly defined. While systemic estrogen exposure and testosterone suppression through FHT profoundly alters circulating sex hormone concentrations, its impact on mucosal microbial composition in TGW remains understudied(15, 16). Geographic variation in diet, antibiotic exposure, sexual health practices, and access to gender-affirming care can also substantially shape mucosal microbial communities(17–19), potentially obscuring or confounding hormone-related signals. *Prevotella* enrichment has been observed among MSM in both the United States and Europe(20–23) but has not been assessed in TGW or across broader geographic contexts. Understanding whether gender identity-associated microbial phenotypes persist across such contexts is essential for defining biologically conserved features of mucosal immunity relevant to HIV prevention.

To address these knowledge gaps, we conducted a multi-site study of individuals assigned male at birth: cisgender MSM and TGW using FHT who have sex with men in Atlanta, Georgia, USA and Bangkok, Thailand. Using cross-sectional and longitudinal study designs, we investigated how gender identity, geography, and feminizing hormone therapy associate with rectal mucosal microbiota composition using novel statistical techniques that focus on driver taxa rather than individual taxa differences that can be difficult to interpret. We further characterized neovaginal microbial communities in a subset of TGW. By integrating behavioral, hormonal, and microbiota data, this study aimed to delineate biological and contextual factors shaping mucosal microbial phenotypes that may underlie HIV vulnerability in gender and geographically diverse populations.

## METHODS

### The clinical cohort.

**Figure 1:**
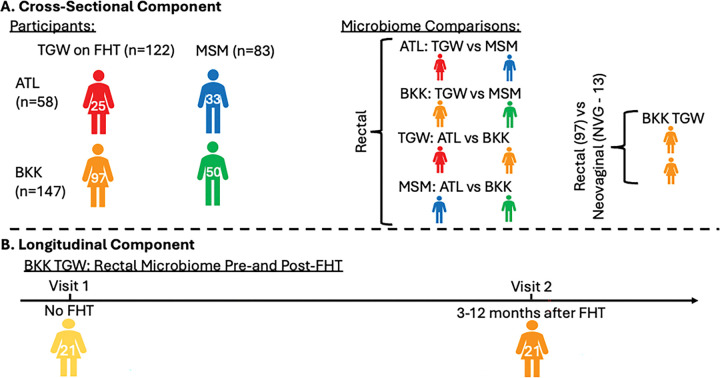
Study Design. We compared the mucosal microbiotas in key populations using two complementary observational study designs – cross-sectional and longitudinal. (A) For the cross-sectional component, we compared the rectal microbiota between transgender women (TGW) and cisgender men who have sex with men (MSM) in Atlanta (ATL); TGW and MSM in Bangkok (BKK); TGW in ATL versus BKK; and MSM in ATL versus BKK. We also compared the rectal and neovaginal microbiota in a subset of TGW in BKK. (B) For the longitudinal component, we compared the rectal microbiota before and 3–12 months after feminizing hormone therapy (FHT) initiation in 21 TGW from BKK.

Cisgender men who have sex with cisgender men (MSM) and transgender women who have sex with men (TGW) aged 18–59 years without HIV and in good health were recruited in Atlanta, Georgia, USA (ATL) and Bangkok, Thailand (BKK) and enrolled between October 2020 and April 2024. The study employed both cross-sectional and longitudinal designs (Fig. 1). The study was approved by the Emory University IRB (CR005-IRB00112414) and the Bamrasnaradura Infectious Diseases Institute IRB (Code P006h/63). All procedures adhered to institutional and international ethical standards, and informed consent was obtained in accordance with the declaration of Helsinki.

### Cross-sectional study

A total of 145 TGW on feminizing hormone therapy (45 ATL; 100 BKK) and 94 MSM (44 ATL; 50 BKK) were enrolled. TGW participants were required to have used FHT (oral, patch, topical, or injection estrogen in any dosage with or without anti-androgens and/or progestin) for at least six months and with stable dosing for at least three months prior to blood and rectal mucosal sampling. All participants reported lifetime receptive anal intercourse (RAI), which is known to influence the rectal microbiota(8, 23–25). In ATL, 13 TGW and 9 MSM did not complete the study; all BKK participants completed the study. Low-quality rectal microbiota samples precluded analysis in 2 ATL TGW, 1 ATL MSM, and 3 BKK TGW. An additional 5 ATL TGW and 1 ATL MSM samples were collected after the final sequencing batch was completed and were thus excluded. Final analyses reported here therefore included 25 ATL TGW, 33 ATL MSM, 97 BKK TGW, and 50 BKK MSM.

Rectal mucosal tissue and blood immune cell subsets, as well as rectal mucosal biopsies for HIV explant challenges, were also collected; those findings are reported separately. Use of synthetic estradiol formulations for FHT is common in Bangkok but uncommon in the United States(26). Across both cohorts, we classified 17β-estradiol gel, estradiol benzoate, estradiol hemihydrate, and estradiol valerate as 17β estradiol formulations and ethinyl estradiol and mestranol as synthetic estradiol formulations. 17β estradiol formulations are detectable by the standard serum estradiol assays used in this study, while synthetic formulations typically are not detected by these assays(8, 23–25).

### Longitudinal study

The longitudinal portion of the study was conducted in BKK only and enrolled 25 TGW planning to initiate FHT within six months who were either FHT-naïve or had discontinued FHT at least six months prior. Three participants were lost to follow-up and one withdrew consent, leaving 21 included in analyses here.

### Study Procedures

#### Cross-sectional study

Participants completed two visits. During the screening visit, eligible participants provided informed consent and then underwent sexual and medical history, medication review, physical examination, rapid HIV testing, and blood collection for safety assessment. The blood and mucosal sampling visit occurred three days to 12 weeks later. Rectal mucosal (RM) secretions were collected using polyurethane swabs inserted via disposable sigmoidoscope or anoscope. Serum estradiol and total testosterone concentrations were measured. TGW who had undergone vaginoplasty were offered optional neovaginal sampling using a speculum and polyurethane swab to collect secretions.

#### Longitudinal study

Participants completed three visits. During the screening visit, informed consent and baseline assessments were performed as above. The pre-FHT sampling visit occurred one day to two weeks later, with RM sampling and serum hormone measurement as above. The post-FHT initiation visit occurred three to 12 months after FHT initiation and repeated the sampling procedures of the prior visit.

#### Serum hormone measurements

Serum estradiol and testosterone concentrations were measured at all sampling study visits in the cross-sectional and longitudinal cohorts. Detailed serum hormone measurement procedures are provided in the **Supplemental Methods**.

#### Microbiota sequencing.

RM swabs were collected once from 205 participants in the cross-sectional study and twice in 21 longitudinal participants before and after FHT initiation. Neovaginal swabs were collected in a subset of 13 BKK TGW participants from the cross-sectional study. To minimize potential for batch effect, all specimens were extracted at Emory University, and sequencing was conducted in two batches that contained roughly equal distributions of MSM, TGW, ATL, and BKK participants. DNA was extracted with the Qiagen DNeasy PowerSoil Pro Kit. The V3/V4 hypervariable regions of the 16S rRNA gene were amplified by real-time PCR and sequenced on an Illumina MiSeq. Detailed sequencing procedures are provided in the **Supplemental Methods**.

#### Microbiota statistical analyses.

Using linear decomposition modeling(1) and BOUTH analyses(2) described further below, RM microbiota were compared by gender identity (ATL TGW vs MSM; BKK TGW vs MSM) and geography (TGW ATL vs BKK; MSM ATL vs BKK) for the cross-sectional study. Rectal and neovaginal microbiotas were also compared among a subset of BKK TGW. For the longitudinal study, RM microbiota before and after FHT initiation were compared. We employed complementary LDM and BOUTH approaches in this study to both identify highly significant correlations with individual taxa and variables of interest using a statistically rigorous method (LDM) and to attempt to identify which among these individual taxa were the most significant contributors to the differences seen using BOUTH.

Microbiota composition was assessed globally and at individual taxon levels. Alpha diversity (Shannon index) was compared using Wilcoxon tests. Relative abundance and presence-absence data were analyzed using linear decomposition modeling (LDM), which is a robust statistical approach that allows the inclusion of multiple study groups and visits into a single model, controls for multiple comparisons, and is a single analysis pathway that combines both global test of effect as well as tests of individual outcomes(1, 27). Presence-absence analyses were included to ensure the detection of rare difference in the rectal microbiome, as they are the preferred method of rare taxa detection(27). Analyses were conducted at the genus level.

We next applied BOUTH analysis (Bottom-Up Approach to Testing Hypotheses) to the cross-sectional relative abundance data to identify the “driver taxa” contributing most strongly to group differences. Bottom-up approaches are better-suited to detecting signals shown within the phylogenetic tree, as microbiome data tend to fall at varying levels of taxonomic depth. BOUTH evaluates associations starting at the lowest phylogenetic level and aggregates evidence upward to identify the taxonomic groups that most influence community structure while controlling a novel error rate called the false selection rate. The direction of effect may be different for each of the lower-level taxa that together contribute to the enriched signal, but we were able to estimate the overall direction of effect using relative abundance LDM at the enriched phylogenetic level. This framework improves interpretability by highlighting biologically meaningful driver taxa rather than large sets of individually significant but difficult-to-contextualize associations(2).

Finally, LDM was conducted at the family level, which had fewer unidentified taxa than the genus level, to explore associations between serum estradiol and testosterone concentrations and microbiome composition. Because no significant associations were identified by LDM, we next used Spearman correlations at the family level that were not corrected for multiple comparisons to assess associations for exploratory/hypothesis generating purposes.

Global significance was defined at p < 0.05, with FDR of 5% for individual taxa, in LDM analyses. Additional details on LDM and presence-absence analyses are provided in the **Supplemental Methods.**

## RESULTS

### The clinical cohort

Demographic and clinical characteristics are summarized in Table 1. The median age was higher in ATL MSM (34 years) than among BKK MSM and TGW in both cities (26 years). The ATL population was racially diverse and representative of the local community. PrEP use was highest in ATL MSM (60.6%) compared with ATL TGW (24%), BKK MSM (32%), and BKK TGW (10.3%). Recent RAI was most common in BK TGW followed by BK MSM, ATL MSM, and ATL TGW). Douching before sex was reported more commonly in BKK participants (TGW 86.6%, MSM 92%) than ATL participants (TGW 56%, MSM 60.6%). Tobacco, alcohol, and drug use were also more frequently reported in ATL than in BKK participants.

### Feminizing hormone therapy regimens

#### Cross sectional cohort.

FHT regimens and durations are presented in Table 2. Compared with ATL TGW, BKK TGW more frequently used synthetic estradiol (ethinyl estradiol or mestranol) BKK TGW were less likely to use progestins than the ATL TGW, but when comparing TGW who were taking progestins, those from BKK reported a longer duration of therapy. Among ATL TGW, spironolactone was the predominant anti-androgen, whereas all BKK TGW used cyproterone acetate. Progestin formulations included hydroxyprogesterone caproate, progesterone, levonorgestrel, and norethisterone. Cyproterone acetate has some progestin activity, however given that its primary clinical use as an anti-androgen, we categorized it as anti-androgen only.

#### Longitudinal cohort.

FHT regimens and duration are summarized in Table 3. Patterns of synthetic estradiol and anti-androgen use were similar to BKK TGW in the cross-sectional cohort, though only ethinyl estradiol was used in the longitudinal cohort. All anti-androgen formulations were cyproterone acetate, and no participants were taking progestins. Participants completed post-FHT sampling a median of 120 days (IQR 96–182) after estradiol initiation.

### Serum hormone concentrations

**Figure 2:**
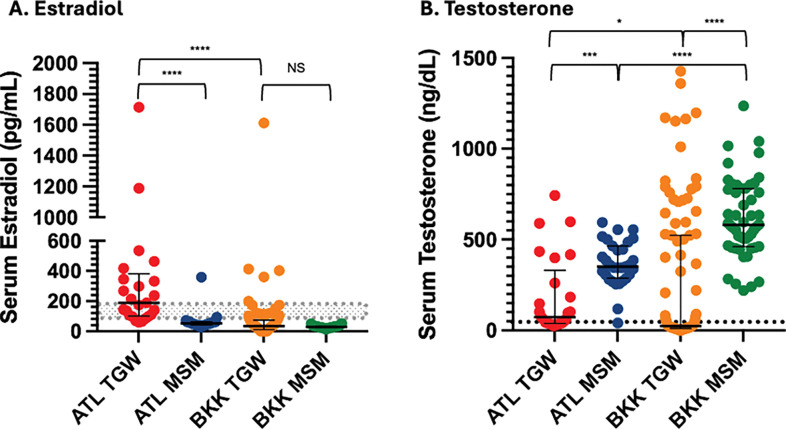
Cross-Sectional Serum Hormone Concentrations. These plots display serum estradiol (A) and testosterone (B) concentration medians and interquartile ranges between the four cross-sectional study groups: ATL TGW (n=25); ATL MSM (n=33); BKK TGW (n=97); BKK MSM (n=50). The shaded area (estradiol) and dotted line (testosterone) indicate therapeutic concentrations for TGW per Endocrine Society Guidelines (100–200 pg/mL for serum estradiol and < 50 ng/dL for serum testosterone). Comparisons were conducted with Mann-Whitney tests. * = p < 0.05, ** = p < 0.01, *** = p < 0.001, **** = p < 0.0001.

**Figure 3:**
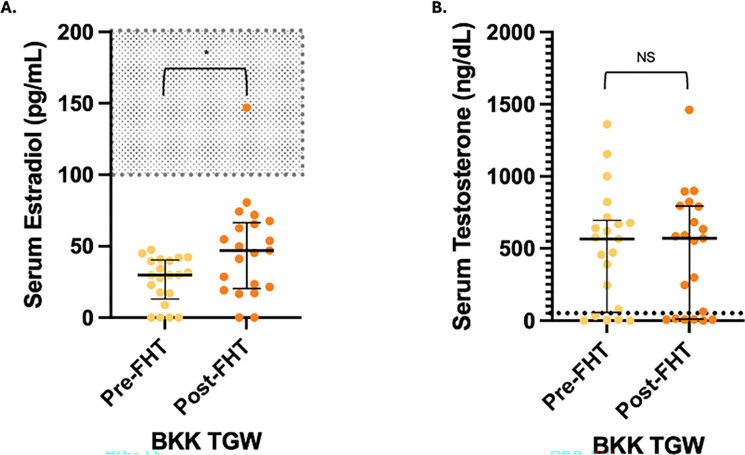
Longitudinal Serum Hormone Concentrations. These plots display serum estradiol (A) and testosterone (B) concentration medians and interquartile ranges in the longitudinal study group before and 3–12 months after feminizing hormone therapy (FHT) initiation (n=21). The shaded area (estradiol) and dotted line (testosterone) indicate therapeutic concentrations for TGW per Endocrine Society Guidelines (100–200 pg/mL for serum estradiol and < 50 ng/dL for serum testosterone). Comparisons were conducted with Mann-Whitney tests. * = p < 0.05.

Cross-sectional serum hormone concentrations are shown in Figure 2. Median estradiol was significantly higher in ATL TGW than BKK TGW, while testosterone was suppressed in both cross-sectional groups of TGW. Longitudinal serum hormone concentrations are shown in Figure 3. While median estradiol increased significantly (p=0.02) from pre-FHT (29.8 pg/mL) to post-FHT (46.9 pg/mL), the effect size was small, and the post-FHT median estradiol remained well below the lower limit of the therapeutic window for this hormone (100 – 200 pg/mL). Median testosterone concentration did not change significantly after FHT initiation and remained persistently above the therapeutic window (<50 ng/dL)(28).

### Rectal microbiota differences by gender identity and geography

Table 4 summarizes mucosal microbiota alpha and beta diversity comparisons.

#### Atlanta:

**Figure 4:**
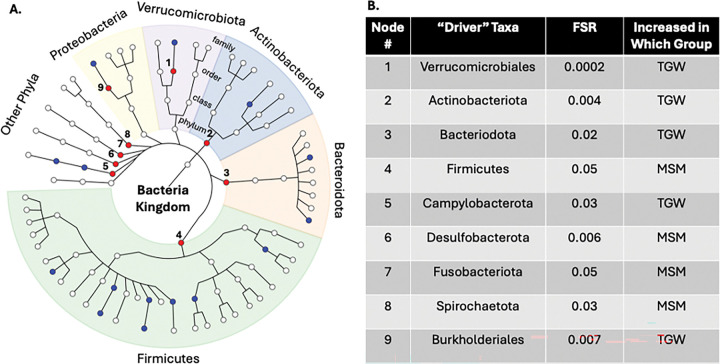
“Drivers” of Gender Identity Differences in Atlanta. We first conducted linear decomposition modeling (LDM) of the rectal microbiota relative abundance data comparing ATL TGW (transgender women in Atlanta; n=25) and ATL MSM (cisgender men who have sex with men in Atlanta; n=33) at the genus level. This analysis demonstrated a global significant difference (p=0.004). We then used the Bottom-Up Approach to Testing Hypotheses (BOUTH) analysis of this LDM data to identify the “driver taxa” (false selection rate, or FSR, <0.05), which represent the highest-level taxa below which there are dense association signals. (A) Demonstrates the BOUTH plot with the nine driver taxa numbered and represented with red circles and significantly different non-driver taxa represented with blue circles. The circle in the middle of the plot represents the *Bacteria* kingdom, and each concentric circle of taxa represents the subsequent phylogenetic level (phylum, class, order, family from center to periphery). The most abundant five phyla are identified with different colors and labeled around the circumference of the circle. (B) Lists the nine “driver taxa” for the significant difference between ATL TGW and ATL MSM, the FSR, and the direction of effect.

TGW and MSM had significantly different rectal microbiota compositions (LDM global p=0.004 for relative abundance; LDM global p=0.0002 for presence-absence). Twenty-one taxa differed by relative abundance and 19 by presence-absence (FDR<5%; **Supplemental Figure 1**). BOUTH analysis (Figure 4) identified “driver taxa” including seven phyla: *Actinobacteriota, Bacteroidota,* and *Campylobacterota* (enriched in TGW) and *Firmicutes, Desulfobacterota, Fusobacteriota,* and *Spirochaetota* (enriched in MSM); and two orders: *Verrucomicrobiales* and *Burkholderiales* (both enriched in TGW). Alpha diversity did not differ by gender identity in ATL (p=0.12).

#### Bangkok:

**Figure 5:**
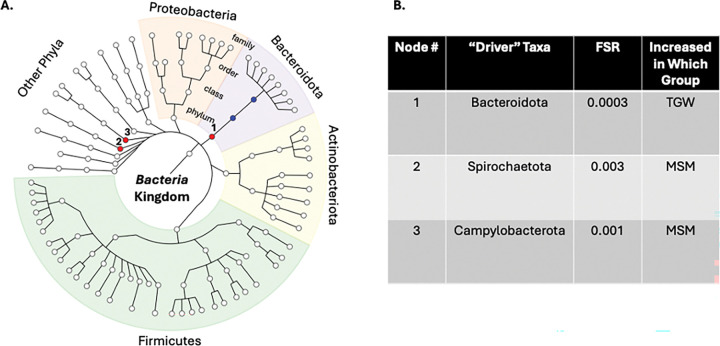
“Drivers” of Gender Identity Differences in Bangkok. We first conducted linear decomposition modeling (LDM) of the rectal microbiota relative abundance data comparing BKK TGW (transgender women in Bangkok; n=97) and BKK MSM (cisgender men who have sex with men in Bangkok; n=50) to the genus level. This analysis demonstrated a significant global difference (p=0.005). We then used the Bottom-Up Approach to Testing Hypotheses (BOUTH) analysis of this LDM data to identify the “driver taxa” (false selection rate, or FSR, <0.05), which represent the highest-level taxa below which there are dense association signals. (A) Demonstrates the BOUTH plot with the three driver taxa numbered and represented with red circles and significantly different non-driver taxa represented with blue circles. The circle in the middle of the plot represents the*Bacteria* kingdom, and each concentric circle of taxa represents the subsequent phylogenetic level (phylum, class, order, family from center to periphery). The most abundant five phyla are identified with different colors and labeled around the circumference of the circle. (B) Lists the three “driver taxa” for the significant difference between BKK TGW and BKK MSM, the FSR, and the direction of effect.

TGW and MSM also showed significant global differences (LDM global p=0.005 for relative abundance, LDM global p=0.03 for presence-absence p=0.03). No individual taxa reached FDR<5%. In exploratory analyses utilizing a more liberal FDR of < 20%, three taxa differed in relative abundance and four in presence-absence (**Supplemental Figure 2**). BOUTH identified three driver phyla: *Bacteroidota* (again enriched in TGW) and *Spirochaetota* and *Campylobacterota* (enriched in MSM; Figure 5). Alpha diversity did not differ by gender identity in BKK (p=0.96).

#### Transgender women:

**Figure 6:**
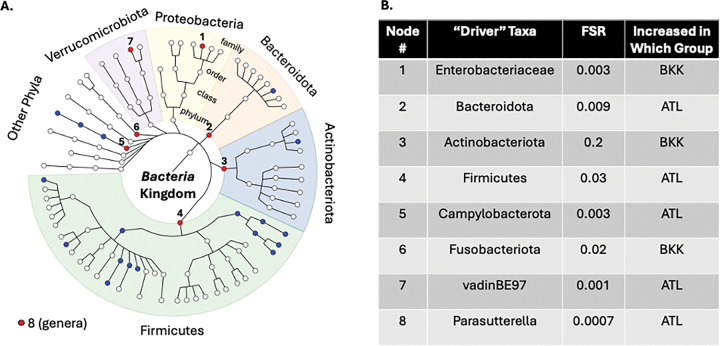
“Drivers” of Location Differences within Transgender Women. We first conducted linear decomposition modeling (LDM) of the rectal microbiota relative abundance data comparing BKK TGW (transgender women in Bangkok; n=97) and ATL TGW (transgender women in Atlanta; n=25) to the genus level. This analysis demonstrated a significant global difference (p=0.002). We then used the Bottom-Up Approach to Testing Hypotheses (BOUTH) analysis of this LDM data to identify the “driver taxa” (False selection rate, or FSR, <0.05), which represent the highest-level taxa below which there are dense association signals. (A) Demonstrates the BOUTH plot with the eight driver taxa numbered and represented with red circles and significantly different non-driver taxa represented with blue circles. The circle in the middle of the plot represents the *Bacteria*kingdom, and each concentric circle of taxa represents the subsequent phylogenetic level (phylum, class, order, family from center to periphery). The most abundant five phyla are identified with different colors and labeled around the circumference of the circle. (B) Lists the eight “driver taxa” for the significant difference between BKK TGW and ATL TGW, the FSR, and the direction of effect.

Rectal microbiota differed significantly between TGW in ATL and BKK (LDM global p=0.002 for relative abundance, LDM global p=0.0006 for presence-absence). Twenty taxa differed by relative abundance and eight by presence-absence (FDR<5%; **Supplemental Figure 3**). BOUTH identified eight driver taxa: five phyla (*Bacteroidota, Firmicutes, Campylobacterota* enriched in ATL TGW (which were also enriched in ATL MSM, discussed below); *Actinobacteriota, Fusobacteriota* enriched in BKK TGW), two families (*vadinBE97* enriched in ATL TGW; *Enterobacteriaceae* enriched in BKK TGW (which was also enriched in BKK MSM, discussed below), and one genus (*Parasutterella,* enriched in ATL TGW; Figure 6). Alpha diversity did not significantly differ by study site among TGW (p=0.052).

#### Cisgender MSM:

**Figure 7:**
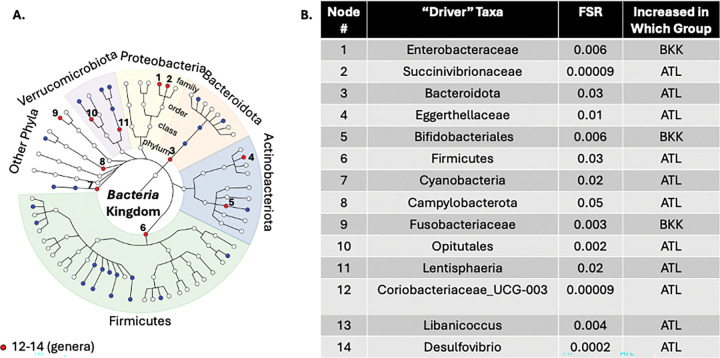
“Drivers” of Location Differences within Cisgender Men who Have Sex with Men. We first conducted linear decomposition modeling (LDM) of the rectal microbiota relative abundance data comparing BKK MSM (cisgender men who have sex with men in Bangkok; n=50) and ATL MSM (cisgender men who have sex with men in Atlanta; n=33) to the genus level. This analysis demonstrated a significant global difference (p=0.0002). We then used the Bottom-Up Approach to Testing Hypotheses (BOUTH) analysis of this LDM data to identify the “driver taxa” (False selection rate, or FSR, <0.05), which represent the highest-level taxa below which there are dense association signals. (A) Demonstrates the BOUTH plot with the 14 driver taxa numbered and represented with red circles and significantly different non-driver taxa represented with blue circles. The circle in the middle of the plot represents the *Bacteria* kingdom, and each concentric circle of taxa represents the subsequent phylogenetic level (phylum, class, order, family from center to periphery). The most abundant five phyla are identified with different colors and labeled around the circumference of the circle. (B) Lists the 14 “driver taxa” for the significant difference between BKK MSM and ATL MSM, the FSR, and the direction of effect.

Rectal microbiota also differed significantly between MSM in ATL and BKK (LDM global p=0.0002 for both relative abundance and presence-absence). Sixty-six taxa differed in relative abundance and 64 in presence-absence between MSM in ATL and BKK (FDR<5%; **Supplemental Figure 4**). BOUTH identified 14 driver taxa: four phyla (*Bacteroidota, Firmicutes, Campylobacterota, Cyanobacteria –* all enriched in ATL MSM); one class (*Lentisphaeria*, enriched in ATL MSM); two orders (*Opitutales,* enriched in ATL MSM; *Bifidobacteriales,* enriched in BKK MSM); four families (*Eggerthellaceae* – enriched in ATL MSM; *Succinivibrionaceae, Fusobacteriaceae, Enterobacteraceae* – enriched in BKK MSM (*Enterobacteraceae* also enriched in BKK TGW as above)); and three genera (*Coriobacteriaceae_UCG-003, Libanicoccus, Desulfovibrio* – all enriched in ATL MSM; Figure 7). Alpha diversity was higher in ATL MSM compared to BKK MSM (p=0.002).

### *Prevotellaceae* and *Bacteroidaceae* enrichment patterns

**Figure 8:**
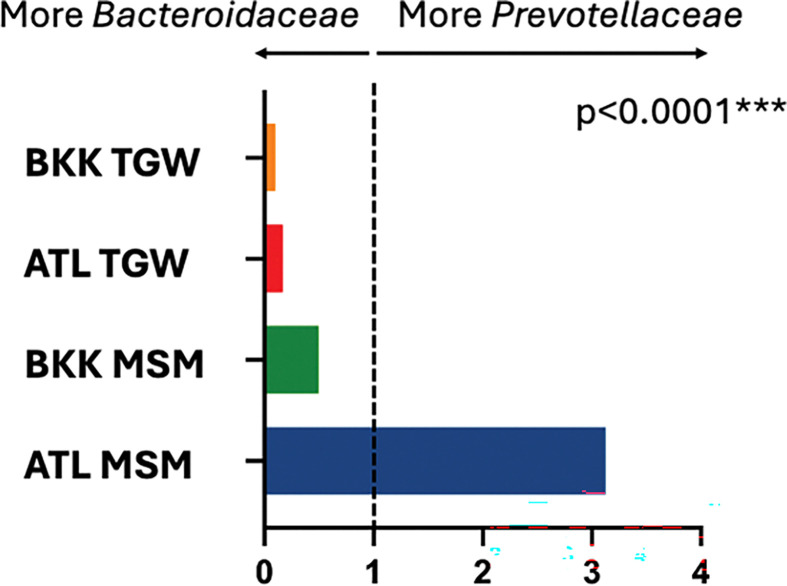
Rectal *Prevotellaceae* to *Bacteroidaceae* abundance ratios in key populations. Rectal *Prevotellaceae*to *Bacteroidaceae* ratios were found to be significantly different between the four groups (p<0.0001 by Kruskal-Wallis). BKK TGW = transgender women in Bangkok; ATL TGW = transgender women in Atlanta; BKK MSM = cisgender men who have sex with men in Bangkok; ATL MSM = cisgender men who have sex with men in Atlanta. The *Prevotellaceae:Bacteroidaceae* ratio was >1 for MSM in ATL but were <1 for MSM in BKK as well as TGW in both ATL and BKK.

To assess whether the pattern of *Prevotellaceae:Bacteroidaceae* enrichment seen in American and European MSM is generalizable to TGW and Asian cohorts, we compared the median *Prevotellaceae:Bacteroidaceae* abundance ratio across the four cross-sectional groups (BKK TGW, ATL TGW, BKK MSM, ATL MSM). Ratios differed significantly across the four groups (Kruskal-Wallis p<0.0001). The median ratio was >1 in ATL MSM but <1 in BKK MSM and in TGW from both sites (Figure 8).

### Rectal and neovaginal microbiota composition

**Figure 9:**
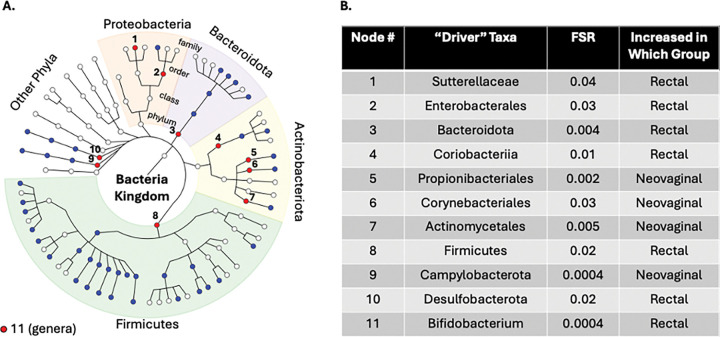
“Drivers” of Rectal versus Neovaginal Mucosal Site Differences. We first conducted linear decomposition modeling (LDM) of the relative abundance data comparing the rectal and neovaginal microbiotas in a subset of BKK TGW (transgender women in Bangkok; n=97 with rectal sampling; n=13 with neovaginal sampling) to the genus level. This analysis demonstrated a significant global difference (p=0.0002). We then used the Bottom-Up Approach to Testing Hypotheses (BOUTH) analysis of this LDM data to identify the “driver taxa” (False selection rate, or FSR, <0.05), which represent the highest-level taxa below which there are dense association signals. (A) Demonstrates the BOUTH plot with the 11 driver taxa numbered and represented with red circles and significantly different non-driver taxa represented with blue circles. The circle in the middle of the plot represents the *Bacteria* kingdom, and each concentric circle of taxa represents the subsequent phylogenetic level (phylum, class, order, family from center to periphery). The most abundant five phyla are identified with different colors and labeled around the circumference of the circle. (B) Lists the 11 “driver taxa” for the significant difference between the rectal and neovaginal microbiotas, the FSR, and the direction of effect.

We next compared the rectal (n=97) and neovaginal (n=13) microbiota in BKK TGW. Relative abundance and presence-absence both differed significantly (LDM global p=0.0002 for each). Eighty-eight taxa differed by relative abundance and 86 by presence-absence (FDR<5%; **Supplemental Figure 5** for taxa enriched in the neovagina and **Supplemental Figure 6** for taxa enriched in the rectum). BOUTH identified 11 driver taxa: four phyla (*Bacteroidota, Firmicutes, Desulfobacterota* – enriched in the rectum; *Campylobacterota* – enriched in the neovagina); one class (*Coriobacteriia* – enriched in the rectum); four orders (*Enterobacterales* – enriched in the rectum; *Propionibacteriales, Corynebacteriales, Actinomycetales* – enriched in the neovagina); one family (*Sutterellaceae* – enriched in the rectum); and one genus (*Bifidobacterium* – enriched in the rectum; Figure 9). Alpha diversity was higher in the rectum compared to the neovagina (p=0.0004).

Among participants with known neovaginal surgical technique (n=12), nine had penile inversion and one had sigmoid colon pull-through. No significant differences in microbiota composition were observed between surgical types (LDM global p=0.3).

### Associations between FHT and the rectal microbiota

We finally assessed associations with FHT and the rectal microbiota composition in two ways: first, by assessing for microbiota composition differences before and after FHT initiation in our longitudinal cohort, and second, and by correlating serum hormone concentrations with microbiota relative abundance in our cross-sectional cohort.

No significant changes were observed before versus after FHT initiation for relative abundance, presence-absence, or alpha diversity (p=0.8, 0.8, and 1, respectively).

Twenty-two percent of the cross-sectional BKK TGW participants were taking synthetic estradiol, which is not detected by clinical serum estradiol assays. Consistent with this, participants on synthetic estradiol had lower measured estradiol concentrations than those on 17β estradiol formulations (Mann-Whitney p=0.001; **Supplemental Figure 7**). Hence, we excluded these participants from the LDM assessing an association between serum estradiol and family-level microbiome relative abundance. This LDM did not show a significant association (LDM global p=0.6). The LDM assessing an association between serum testosterone and family-level microbiome relative abundance also did not show a significant association (LDM global p=0.6). We subsequently conducted Spearman correlation measurements between serum hormone concentrations and family-level microbiome relative abundance to detect more subtle potential associations for hypothesis-generating purposes. These showed significant (p<0.05) correlations between serum estradiol and six families and between serum testosterone and five families (**Supplemental Tables 1 and 2**).

## DISCUSSION

Here we have characterized rectal and neovaginal mucosal microbiota phenotypes in TGW and MSM in both Atlanta and Bangkok. We found highly significant rectal microbiota differences associated with both gender and geography. We used a novel statistical approach, the BOUTH method, to distinguish “driver taxa” amidst large numbers of significant individual taxa by LDM that were the most important contributors to cross-sectional group differences. This further enabled identification of (1) microbiota characteristics by gender identity that persist across geographic locations and (2) characteristics by location that persist across gender identity. Finally, we have expanded on limited existing literature describing the neovaginal microbiota and have identified enrichment of the BASIC taxa, a cluster of anaerobic taxa that have strong associations with HIV seroconversion in the penile and rectal mucosa(6, 29).

BOUTH analysis offers advantages over traditional microbiota association methods by explicitly leveraging the hierarchical structure of microbial taxonomy(2). Instead of testing each taxon independently or collapsing signals without regard to phylogenetic relatedness, BOUTH begins at the lowest taxonomic level for which individual tests are available and synthesizes evidence as it moves upward through the taxonomic tree, enabling detection of higher-order taxa that harbor dense and biologically coherent association signals. This bottom-up strategy controls the false selection rate, yielding results that are statistically robust and easier to interpret within ecological and evolutionary contexts. Simulation studies and empirical applications consistently show that BOUTH more effectively isolates true “driver taxa” than conventional methods, which often overlook tree-structured dependencies or fail to aggregate related signals. In our study, BOUTH allowed us to distill numerous significant associations detected by the LDM into a focused set of driver taxa that most strongly accounted for observed differences by gender identity and location. Through this approach, we identified several driver taxa that persisted across contexts: *Bacteroidota*, which was enriched in TGW at both locations; *Enterobacteriaceae*, which was enriched in both TGW and MSM in BKK; and *Bacteroidota, Firmicutes, and Campylobacterota*, which were all enriched in both TGW and MSM in ATL. These findings highlight phylogenetically coherent microbial signatures that may underlie geographic- and gender-associated mucosal phenotypes.

We identified rectal microbiota differences influenced by geographic location that persisted across gender identities. *Enterobacteriaceae* was enriched in both TGW and MSM in Bangkok compared to Atlanta. This enrichment has been associated with dysbiosis, antibiotic exposure, and urbanization, consistent with prior studies demonstrating high community carriage of ESBL-producing *Enterobacteriaceae* in Thailand linked to widespread antibiotic use(30). Conversely, the rectal microbiota in Atlanta showed enrichment for *Bacteroidota, Firmicutes*, and *Campylobacterota*, which are associated with Western dietary patterns rich in animal proteins, fats, and fiber(31). These findings underscore the importance of geographic context in microbiota analyses.

Within both Atlanta and Bangkok, gender identity was associated with striking differences in rectal microbiota composition, most prominently at the phylum level. Our BOUTH analysis revealed that *Bacteroidota* enrichment in the rectal microbiota of TGW in both locations was a “driver” of the differences between the rectal microbiota in TGW and MSM. *Bacteroidota* is a large phylum with diverse roles in gut homeostasis; notably, many taxa within this group express β-glucuronidase enzymes that participate in estrogen metabolism by deconjugating estrogens otherwise destined for excretion(32). This reactivation enables enterohepatic recycling of estrogens. *Bacteroidota* has been previously associated with exogenous estradiol use; it was found to be enriched in the gut microbiota in postmenopausal women taking hormone therapy (estradiol +/− progesterone) compared to those not taking hormone therapy in a recent study(33). *Firmicutes* was also identified as a driver of rectal microbiota differences in ATL MSM compared to ATL TGW and was enriched in ATL MSM. A recent systematic review demonstrated that women with higher serum estradiol concentrations had enrichment for *Bacteroidota* when compared to *Firmicutes*, which is consistent with *Bacteroidota* enrichment in TGW and *Firmicutes* enrichment in ATL MSM in our study being potentially estradiol-associated(9). At the genera level, our LDM results also showed enrichment of taxa associated with estrogen metabolism, such as *Bacteroides* and *Ruminococcus gnavus*, in TGW compared to MSM across both sites which further suggests that FHT may exert effects on the rectal microbiota that transcend geography(34). *Ruminococcus gnavus* has been associated with endogenous estrogen in cisgender women, supporting this link(9). In our study, the median serum estradiol concentration was within the therapeutic range for ATL TGW but not for BKK TGW. This may have contributed to the less-striking differences that were seen between BKK MSM and BKK TGW when compared with the differences seen within the two gender identities in Atlanta.

While serum estradiol concentrations were therapeutic only among TGW in ATL, median serum testosterone concentrations were consistently suppressed across TGW in both ATL and BKK. This makes testosterone suppression the only FHT-related effect uniformly present in our cohort. Notably, the taxa associated with testosterone suppression in our study did not overlap with those reported in the only other longitudinal analysis of FHT in TGW(35). This is likely because participants in that study were uniformly receiving therapeutic estradiol, which confounds the effects of testosterone suppression. This may also be because that study profiled stool rather than rectal mucosal communities, which can be subtly different. Likewise, our findings did not overlap with taxa linked to androgen deprivation therapy in cisgender men with prostate cancer(36), a population in whom disease state and treatment likely exert substantial additional microbiota pressures. Taken together, these discrepancies suggest that the rectal microbiota signatures we observe in TGW reflect effects of testosterone suppression that are distinct from those seen in cisgender man and cannot be inferred from studies combining estradiol supplementation or different anatomic niches. Given testosterone’s immunomodulatory properties, these relationships may also reflect indirect interactions with local immune activity in the rectal mucosa.

Despite observing hormone-associated and gender identity-linked differences, we did not detect significant changes in rectal microbiota composition before and after FHT initiation in our longitudinal cohort. Several factors may explain this discrepancy. First, most participants did not reach therapeutic hormone levels at the second sampling visit. Median serum estradiol levels remained well below the therapeutic window of 100–200 pg/mL at both pre- and post-FHT visits. Serum testosterone concentrations remained well above the suppression goal of < 50 ng/dL; they actually increased slightly from pre-FHT to post-FHT. This is overall consistent with known common use of over-the-counter hormone regimens, including oral contraceptives, for FHT in Bangkok, a practice that frequently results in suboptimal sex hormone concentrations(26). While the largest recent study assessing the impact of therapeutic FHT on the gut microbiota in cisgender women identified microbiota changes at six month follow up(37), another recent longitudinal study assessing the impact of oral contraceptive pills (which are typically formulated with synthetic estradiols) in cisgender women did not see significant changes in the gut microbiome before or at either one or six months after initiating therapy(38). Microbial communities often respond gradually to endocrine perturbations, suggesting that longer-duration FHT may be necessary to observe the full magnitude of microbiota remodeling, particularly once stable therapeutic hormone concentrations are achieved. 16S rRNA sequencing may also have limited our ability to detect subtle changes. A recent metagenomic study of German TGW found modest changes 12 weeks after endocrinologist-prescribed FHT initiation, including altered abundance of *Parabacteroides goldsteinii* and *Escherichia coli*, suggesting both that therapeutic, physician-prescribed FHT may induce rectal microbiota changes and that metagenomic approaches may capture effects missed here(35). Thus it is plausible that therapeutic, physician-prescribed hormonal exposure combined with a longer median follow up time and/or metagenomic sequencing might have revealed microbiota shifts that were not seen in our study.

Prior studies conducted in the United States have reported enrichment of the *Prevotellaceae* family when compared to the *Bacteroidaceae* family in MSM having receptive anal intercourse(8), and several subsequent studies in the United States and Europe demonstrated enrichment of the *Prevotella* genus (a member of the *Prevotellaceae* family) when compared to the *Bacteroides* genus (a member of the *Bacteroidaceae* family)(20–23). Our study expanded on these data, as these ratios have not been previously assessed in Asian populations or in transgender women. We observed *Prevotellaceae* enrichment only among MSM in Atlanta. TGW at both sites and MSM in Bangkok to a lesser degree exhibited *Bacteroidaceae* enrichment, suggesting that *Prevotellaceae* enrichment may be context-specific and influenced by geography and gender identity in addition to sexual practice.

The populations in Atlanta and Bangkok, and across gender identities, represent complex phenotypes that cannot be defined by a single variable such as hormone use. Behavioral differences including PrEP use, RAI, lubricant use, douching, and drug use vary significantly between groups. For example, ATL MSM participants had the highest PrEP use, which may explain that group’s enrichment for *Catenibacterium* and *Holdemanella*, taxa previously linked to PrEP exposure(39–41). *Escherichia-Shigella* had increased probability of presence in BK TGW compared to ATL TGW in the cross-sectional LDM. BK TGW had the highest number of recent RAI encounters in our study, and these taxa are sexually transmissible enteric pathogens previously identified among MSM and associated with RAI and oral-anal contact(42, 43). There are also many other behavioral factors, like diet and douching prior to sex, that likely influence the gut microbiota that we were unable to fully characterize in this study. Taken together, hormone use, behavioral factors, and geography combine to generate mucosal microbiota phenotypes that may influence health and disease vulnerability. Despite overlapping sexual practice and shared HIV vulnerability, TGW and MSM exhibit multifaceted biological and behavioral differences including distinct microbiota compositions at mucosal sites that warrant stratified consideration in future HIV prevention studies.

Our work also adds to the limited literature on the neovaginal microbiota. Compared with the rectum, BOUTH analysis demonstrated neovaginal enrichment of *Propionibacteriales, Corynebacteriales*, and *Actinomycetales*, taxa containing aerobic or microaerophilic skin and mucosal commensals. Their increased abundance may reflect greater oxygen exposure of the neovagina, which lacks an external sphincter and often requires dilation. Because 69% of participants underwent penile inversion vaginoplasty, these taxa may also represent persistent penile skin flora. We observed high neovaginal abundance of *Porphyromonas, Peptostreptococcus, Prevotella*, and *Mobiluncus*, consistent with prior studies, as well as numerous taxa not previously described in the neovagina(44). Several of the most enriched genera (*Prevotella, Peptostreptococcus*, and *Dialister*) overlap with the BASIC taxa (Bacterial Associated with Seroconversion, Inflammation, and Immune Cells) which have been linked to mucosal inflammation, epithelial barrier disruption, HIV target cell recruitment, and HIV seroconversion in the penile mucosa(29, 45). The role of the neovagina in HIV acquisition is not well understood. Direct evidence of neovaginal HIV transmission is lacking, in part because multiple exposure routes, including receptive anal intercourse, complicate definitive attribution. Nevertheless, case reports and small series have documented other sexually transmitted infections including chlamydia, gonorrhea, and HPV in the neovagina, supporting the biological plausibility of sexual transmission of pathogens(46–48). Given that both the rectal and vaginal microbiota can influence HIV acquisition(7), it is plausible that the neovaginal microbiota may contribute to some of the elevated HIV vulnerability observed in TGW.

Our study is limited by a modest sample size, particularly with respect to the longitudinal cohort. It is also limited by reliance on 16S microbiota sequencing and subtherapeutic hormone levels in our Bangkok cohorts. Behavioral factors such as PrEP use, douching, STI history, and substance use also differed significantly between groups, complicating isolation of FHT-specific effects. Nonetheless, our design enabled a comprehensive characterization of gender- and geography-associated mucosal microbiota phenotypes. Because mucosal HIV transmission likely reflects the interaction of multiple biological and behavioral factors, the combined microbial signatures described here may represent more realistic translational targets for prevention efforts that individual taxa alone.

In conclusion, this multi-site study used advanced statistical methods including LDM and BOUTH analyses to identify microbiota signatures associated with gender identity that persist across geographic locations. We identified novel signatures associated with testosterone suppression in TGW as well as evidence of enrichment in estrogen-metabolizing taxa in TGW on FHT. We also provide one of the most detailed characterizations to date of the neovaginal microbiota, identifying taxa with potential implications for inflammation and HIV vulnerability. Future work incorporating metagenomics and metabolomics will be critical to defining the functional and immunologic consequences of these microbial differences. Assessing gender identity as a complex biosocial phenotype, and recognizing its unique biological and behavioral correlates, will be essential for developing HIV prevention and health strategies with real-world applicability.

## Supplementary Material

Supplementary Files

This is a list of supplementary files associated with this preprint. Click to download.

• SupplementalInformation.docx

## Figures and Tables

**Table 1:** Demographic data.

	Cross-Sectional Cohort					Longitudinal Cohort
	Atlanta		Bangkok			Bangkok
Characteristic	TGW (n=25)	MSM (n=33)	TGW (n=97)	MSM (n=50)	P value	TGW (n=21)
Age in years; median (IQR)	26 (24–33)	34 (28–38)	26 (23–32)	26 (24–28)	0.0002	28 (24–34)
Race; n (%)						
Black	8 (32)	15 (45.5)	0 (0)	0 (0)	N/A	0 (0)
White	14 (56)	13 (39.4)	0 (0)	0 (0)		0 (0)
Asian	2 (8)	5 (15.2)	97 (100)	50 (100)		21 (100)
Other	1 (4)	0 (0)	0 (0)	0 (0)		0 (0)
# of RAI events in prior 3 months; median (IQR)	1 (0–2)	2 (0.5–4.5)	5 (2–10)	3 (2–8.5)	0.0001	5 (2.25–8.25)
PrEP use; n (%)	6 (24)	20 (60.6)	10 (10.3)	16 (32)	<0.0001	5 (23.8)
Lubricant use; n (%)	22 (88)	30 (90.9)	88 (90.7)	49 (98)	0.27	20 (95.2)
Douche before sex; n (%)	14 (56)	20 (60.6)	84 (86.6)	46 (92)	<0.0001	17 (81.0)
Self-reported prior STI; n (%)	6 (24)	18 (54.6)	9 (9.3)	16 (32)	<0.0001	2 (9.5)
Tobacco in prior 6 months; n (%)	9 (36)	9 (27.3)	12 (12.4)	6 (12)	0.02	2 (9.5)
Current alcohol use; n (%)	20 (80)	25 (75.8)	31 (32)	9 (18)	<0.0001	6 (28.6)
Drug use in prior 6 months; n (%)	12 (48)	12 (36.4)	6 (6.2)	0 (0)	<0.0001	2 (9.5)

TGW = transgender women who have sex with men; MSM = cisgender men who have sex with men; RAI = receptive anal intercourse; PrEP = pre-exposure prophylaxis; STI = sexually transmitted infection; IQR = intra-quartile range. Statistical comparisons were made with Kruskal-Wallis test for continuous variables (Age, # of RAI events in prior 3 months) and Fisher’s exact test for discrete variables (Race, PrEP use, Lubricant use, Douche use, Self-reported prior STI, Tobacco use, Alcohol use, Drug use).

**Table 2:** Feminizing hormone therapy (FHT) regimens and durations for the Cross-Sectional Cohort.

	Cross-Sectional Cohort		
FHT Regimen	Atlanta TGW (n=25)	Bangkok TGW (n=97)	P value
Synthetic estradiol; n (%)	0 (0)	22 (22.7)	0.03
Months on estradiol; median (IQR)	20.5 (12–37)	85 (41–120)	0.0002
Anti-androgen; n (%)	18 (72)	65 (67)	<0.0001
Months on anti-androgen; median (IQR)*	17 (12–27)	79 (42–109)	0.15
Progestin; n (%)	11(44)	10 (10.3)	0.007
Months on progestin; median (IQR)*	12 (5.75–24)	97 (48–143)	0.12

TGW = transgender women who have sex with men; MSM = cisgender men who have sex with men. Statistical comparisons were made with Mann-Whitney test for continuous variables and Fisher’s exact test for discrete variables. Synthetic estradiol formulations included ethinyl estradiol and mestranol. For ATL TGW, spironolactone comprised most anti-androgen formulations, with one participant taking cyproterone acetate. For BK TGW, 100% of anti-androgen formulations were cyproterone acetate. Progestin formulations included hydroxyprogesterone caproate, progesterone, levonorgestrel, and norethisterone.

* For the months on anti-androgen and progestin, we limited analysis to those who reported use of these medications.

**Table 3:** Feminizing hormone therapy (FHT) regimens and durations for the Longitudinal Cohort.

Longitudinal Cohort - Bangkok TGW (n=21)	
FHT Regimen	
Synthetic estradiol; n (%)	4 (19.1)
17β estradiol; n (%)	18 (85.7)
Days between estradiol initiation and post-FHT sampling; median (IQR)	120 (96–182)
Anti-androgen; n (%)	13 (61.9)
Days between anti-androgen initiation and post-FHT sampling; median (IQR)	99 (0–158)
Progestin; n (%)	0 (0)

TGW = transgender women who have sex with men; MSM = cisgender men who have sex with men. 100% of synthetic estradiol formulations were ethinyl estradiol. 17β estradiol formulations include 17β-estradiol gel, estradiol benzoate, estradiol hemihydrate, and estradiol valerate. 100% of anti-androgen formulations were cyproterone acetate.

**Table 4:** Summary of mucosal microbiome comparisons.

Summary of Mucosal Microbiome Comparisons							
		α Diversity		β Diversity			
Type of Comparison	Group Comparison	Median Shannon Index	P value	Relative Abundance LDM global p	# Differentially Abundant Taxa, FDR < 5%	Presence-Absence LDM global p	# Taxa w/ Differential Probability of Presence, FDR < 5%
Gender Identity	ATL: TGW vs MSM	2.82 vs 3.13	0.12	0.004	21 (13 ↑ in MSM; 8 ↑ in TGW)	0.0002	19 (12 ↑ in MSM; 7 ↑ in TGW)
	BKK: TGW vs MSM	2.67 vs 2.61	0.96	0.005	0 (3 w/ FDR <20%; 2 ↑ in MSM; 1 ↑ in TGW)	0.03	0 (4<20%; 3 ↑ in MSM; 1 ↑ in TGW)
Geographic Location	TGW: ATL vs BKK	2.82 vs 2.67	0.052	0.002	20 (16 ↑ in ATL; 4 ↑ in BKK)	0.0006	8 (3 ↑ in ATL; 5 ↑ in BKK)
	MSM: ATL vs BKK	3.13 vs 2.61	0.002	0.0002	66 (55 ↑ in ATL; 11 ↑ in BKK)	0.0002	64 (50 ↑ in ATL; 14 ↑ in BKK)
Mucosal site	BKK TGW: Rectal vs Neovaginal	2.65 vs 2.18	0.0004	0.0002	88 (60 ↑ in rectum; 28 ↑ in neovagina)	0.0002	86 (70 ↑ in rectum; 16 ↑ in neovagina)
Pre/Post FHT	BKK TGW: Pre- vs Post-FHT	2.82 vs 2.61	1	0.8	0	0.8	0

RA = relative abundance analysis. LDM = linear decomposition modeling. FDR = false discovery rate. PA = presence absence analysis. FHT = feminizing hormone therapy. ATL = Atlanta, Georgia, USA. BKK = Bangkok, Thailand. TGW = transgender women who have sex with men. MSM = cisgender men who have sex with men. Median Shannon Indices were compared with a Wilcoxon test.

## Data Availability

The microbiome dataset generated and analyzed during the current study is available upon request to the corresponding author (Vanessa Van Doren – vvandoren@brownhealth.org).
